# Adequacy of Serial Self-performed SARS-CoV-2 Rapid Antigen Detection Testing for Longitudinal Mass Screening in the Workplace

**DOI:** 10.1001/jamanetworkopen.2022.10559

**Published:** 2022-05-06

**Authors:** Jesse Papenburg, Jonathon R. Campbell, Chelsea Caya, Cynthia Dion, Rachel Corsini, Matthew P. Cheng, Dick Menzies, Cédric P. Yansouni

**Affiliations:** 1 Division of Pediatric Infectious Diseases, Department of Pediatrics, Montreal Children’s Hospital, Montreal, Canada; 2 Division of Microbiology, Department of Clinical Laboratory Medicine, Optilab Montreal–McGill University Health Centre, Montreal, Canada; 3 McGill Interdisciplinary Initiative in Infection and Immunity, Montreal, Canada; 4 Department of Epidemiology, Biostatistics, and Occupational Health, School of Population and Global Health, McGill University, Montreal, Canada; 5 Respiratory Epidemiology and Clinical Research Unit, Centre for Outcomes Research and Evaluation, Research Institute of the McGill University Health Centre, Montreal, Canada; 6 Division of Infectious Diseases, Department of Medicine, McGill University Health Centre, Montreal, Canada; 7 J.D. MacLean Centre for Tropical Diseases, McGill University, Montreal, Canada

## Abstract

**Importance:**

Longitudinal mass testing using rapid antigen detection tests (RADT) for serial screening of asymptomatic persons has been proposed for preventing SARS-CoV-2 community transmission. The feasibility of this strategy relies on accurate self-testing.

**Objective:**

To quantify the adequacy of serial self-performed SARS-CoV-2 RADT testing in the workplace, in terms of the frequency of correct execution of procedural steps and accurate interpretation of the range of possible RADT results.

**Design, Setting, and Participants:**

This prospective repeated cross-sectional study was performed from July to October 2021 at businesses with at least 2 active cases of SARS-CoV-2 infection in Montreal, Canada. Participants included untrained persons in their workplace, not meeting Public Health quarantine criteria (ie, required quarantine for 10 days after a moderate-risk contact with someone infected with SARS-CoV-2). Interpretation and performance were compared between participants who received instructions provided by the manufacturer vs those who received modified instructions that were informed by the most frequent or most critical errors we observed. Data were analyzed from October to November 2021.

**Exposures:**

RADT testing using a modified quick reference guide compared with the original manufacturer’s instructions.

**Main Outcomes and Measures:**

The main outcome was the difference in correctly interpreted RADT results. Secondary outcomes included difference in proportions of correctly performed procedural steps. Additional analyses, assessed among participants with 2 self-testing visits, compared the second self-test visit with the first self-test visit using the same measures.

**Results:**

Overall, 1892 tests were performed among 647 participants, of whom 278 participants (median [IQR] age, 43 [31-55] years; 156 [56.1%] men) had at least 1 self-testing visit. For self-test visit 1, significantly better accuracy in test interpretation was observed among participants using the modified quick reference guide than those using the manufacturer’s instructions for reading results that were weak positive (64 of 115 participants [55.6%] vs 20 of 163 participants [12.3%]; difference, 43.3 [95% CI, 33.0-53.8] percentage points), positive (103 of 115 participants [89.6%] vs 84 of 163 participants [51.5%]; difference, 38.1 [95% CI, 28.5-47.5] percentage points), strong positive (219 of 229 participants [95.6%] vs 274 of 326 participants [84.0%]; difference, 11.6 [95% CI, 6.8-16.3] percentage points), and invalid (200 of 229 participants [87.3%] vs 252 of 326 participants [77.3%]; difference, 10.0 [95% CI, 3.8-16.3] percentage points). Use of the modified guide was associated with improvements on self-test visit 2 for results that were weak positive (difference, 15.4 [95% CI, 0.7-30.1] percentage points), positive (difference, 19.0 [95% CI, 7.2-30.9] percentage points), and invalid (difference, 8.0 [95% CI, 0.8-15.4] percentage points). For procedural steps identified as critical for test validity, adherence to procedural testing steps did not differ meaningfully according to instructions provided or reader experience.

**Conclusions and Relevance:**

In this cross-sectional study of self-performed SARS-CoV-2 RADT in an intended-use setting, a modified quick reference guide was associated with significantly improved accuracy in RADT interpretations.

## Key Points

**Question:**

Can untrained persons correctly perform and interpret the results of SARS-CoV-2 rapid antigen detection tests (RADT), and can performance be optimized?

**Findings:**

In this cross-sectional study of 278 participants self-performing SARS-CoV-2 RADT in an intended-use setting, the accuracy of RADT interpretation was poor when the manufacturer’s instructions were used. A modified quick reference guide was associated with significantly better user performance.

**Meaning:**

These findings suggest that longitudinal mass RADT testing for SARS-CoV-2 could be accurately self-performed in an intended-use setting but there are potential interventions to optimize performance.

## Teaser

This cohort study assesses the execution and interpretation of SARS-CoV-2 rapid antigen detection tests when performed by untrained persons an intended-use setting.

## Introduction

Ongoing transmission of SARS-CoV-2 is frequently driven by asymptomatic or presymptomatic individuals.^1,2^ Mass deployment of lateral-flow rapid antigen detection tests (RADT) for serial screening of asymptomatic persons has been proposed for preventing community transmission, with the aim of promptly detecting individuals most likely to be infectious.^3,4,5,6^ This strategy may be even more relevant with the emergence of the Omicron (B.1.1.529) variant^7^ that may evade immunity afforded by vaccination.

The feasibility of longitudinal mass testing relies on implementation of self-performed RADT where people live, work, or attend school. However, several factors impede this deployment. These include regulatory requirements that RADT be performed by trained health care workers in some countries^8^ and lack of data on the accuracy of self-administration in their intended-use settings.^9^ Similarly, to our knowledge, no studies have assessed the impact of repeated lateral-flow testing on user performance.

In this study, we report the results of a prospective field evaluation of serial self-performed RADT for SARS-CoV-2 among untrained, asymptomatic persons in their workplaces. The primary objective was to quantify the adequacy of self-testing, in terms of the frequency of correct execution of procedural steps and the accuracy of interpretation of the range of possible RADT results. We also aimed to compare results using the instructions provided by the manufacturer with results using modified instructions that were informed by the most frequent or most critical errors we observed.

## Methods

This cross-sectional study was approved by the Research Ethics Board of the Research Institute of the McGill University Health Centre. All participants provided written informed consent. The study followed the Strengthening the Reporting of Observational Studies in Epidemiology (STROBE) reporting guideline for cross-sectional studies.

### Study Design and Setting

In this prospective, repeated, cross-sectional study, businesses in Montreal, Canada, with at least 2 active cases of SARS-CoV-2 infection within a 14-day period were identified by the Montreal Department of Public Health. Businesses with newly identified outbreaks were contacted by the study team by telephone and offered participation in the study via the deployment of 1 of 4 mobile teams on-site. Among outbreak businesses, priority was given to contacting those with greater than 50 employees to increase the probability of identifying participants with unsuspected SARS-CoV-2 infection.^10^ For businesses that agreed to participate, study visits were scheduled twice weekly for 2 weeks (4 visits). Study data were collected and managed using REDCap.^11^

Any worker in outbreak businesses who could provide written informed consent was considered eligible to participate. Participants were not compelled by the study team or by their employer to attend all 4 study visits and could withdraw from further testing if they no longer wished to participate.

### Data Collection

Data on self-reported participant age, gender, race and ethnicity, language preference (English or French), COVID-19 symptoms, exposures, and vaccination were collected. Race and ethnicity were self reported and categorized as Asian, Black, Hispanic, Indigenous, mixed, White, and other (including Algerian, Arab, Bulgarian, Canadian, Chilean, Guatemalan, Haitian, Italian, Mediterranean, Moroccan, Peruvian, and Portuguese). Race and ethnicity and other sociodemographic measures were recorded to account for the heterogeneity of SARS-CoV-2 community transmission. Additionally, we assessed testing results, adequacy of performance of test procedures, and result interpretation.

### Diagnostic Testing Procedures

The RADT used was the Panbio COVID-19 Ag Rapid Test Device (Abbott Laboratories). At each visit, trained study personnel instructed participants on procedures to perform self-collected nasal midturbinate swab specimens. On visits 1 and 3, all postspecimen collection steps and result interpretations were performed by study personnel. On visits 2 (first self-testing visit) and 4 (second self-testing visit), these steps were performed by the participant. During self-testing, study personnel assessed 13 RADT procedural steps performed by the participant as correct or incorrect. If any of 6 steps deemed to be critical to testing integrity were assessed as incorrectly performed, the participant was informed at the end of the procedure and testing was redone by study personnel. Participants with a positive RADT result were offered confirmatory laboratory-based polymerase chain reaction (PCR) testing at a local testing center.

### Result Interpretation Using a Proficiency Panel

A proficiency panel of 7 RADT test results was created using serial dilutions of the positive control reagent and aimed to evaluate participants’ ability to interpret the range of possible signal intensities of the test line, including results classified as weak positive, positive, strong positive, invalid, and negative (eFigure 1 in the Supplement). The panel was printed in color at actual size and high resolution on cardboard. At visits 2 and 4, participants were asked to interpret the 7 RADT results, in addition to their own RADT result.

### Intervention

Participants were initially given access to the complete manufacturer’s instructions, the visual quick reference guide (eFigure 2 in the Supplement), and no time limit during self-testing visits. Study personnel did not give any additional guidance. Written authorization from the manufacturer was obtained for reproduction of their quick reference guide in this study. Following a planned interim analysis, we developed a modified quick reference guide (eFigure 3 in the Supplement) that addressed the most frequently observed user errors in RADT procedural steps and interpretation, and we deployed it subsequently.

The salient elements of the modified quick reference guide included the addition of clear and simple text to the pictograms in the manufacturer’s instructions. Critical procedural steps were emphasized with bold red text. These stressed using the correct quantity of buffer to specimen tubes, adding the recommended volume of specimen to the rapid test device, and reading the result at the prescribed time interval. Test interpretation was guided by a series of questions and tailored images: “1. Is the test INVALID?”; “2. Is the test POSTIVE?”; and “3. Is the test NEGATIVE?” Prior to implementation in the study, linguistic elements in both English- and French-language versions of the modified quick reference guide were refined through consultation with colleagues who work mainly with migrants and refugees not fluent in either language.

### Outcomes

Among all participants with at least 1 self-testing visit, we compared the group using our modified quick reference guide with the group using the original manufacturer’s instructions. Among participants with 2 self-testing visits, we compared the second self-test visit with the first self-test visit. For each comparison, our primary outcome was the difference in the proportion of correctly interpreted RADT proficiency panel results. Our secondary outcome was the difference in proportion of correctly performed procedural steps.

### Sample Size Estimate

Recruitment of participants using the manufacturer’s instructions (study phase 1) proceeded until the overall weighted mean of the accurate readings could be estimated with 6.0% precision at the 95% confidence level. A modified quick reference guide informed by the analysis of phase 1 data was expected to yield at least a 12.0–percentage point improvement in RADT reading accuracy vs the manufacturer's instructions. Based on an expected overall weighted mean of the accurate readings of 60.0% (95% CI, 54.0%-66.0%) in phase 1, the number of readings required to achieve an accuracy of 72.0% with 6.0% precision was 216 readings per result category (invalid, negative, weak positive, positive, and strong positive). At the rate of 2 readings per person per visit, this corresponds to a recruitment of 108 people using the modified instructions.

### Statistical Analysis

Baseline characteristics were summarized according to self-testing visit and reference guide type. Estimates of 95% CIs around a proportion or around the difference of 2 proportions were calculated according to a binomial distribution using the Wilson Score method. We used the term *sensitivity* to refer to the proportion of accurately identified RADT result interpretations of positive and of invalid results; *specificity* referred to accurate RADT result interpretation of negative results.

Generalized linear mixed-effects models were fit to evaluate the association of the use of the modified quick reference guide with accurate RADT proficiency panel result interpretation. Outbreak business and participant were modeled as a random intercepts; participant age (in years), gender, RADT result type (positive, strong positive, negative, or invalid) and an interaction term between RADT result type and instruction type were included as covariates (fixed effects).

Data were analyzed using R statistical software version 3.5.2 (R Project for Statistical Computing). Because of the small number of variables to be analyzed and because missingness was no greater than 5%, a complete case analysis was performed for the modeling. Statistical significance was assessed by using 2-tailed tests, with α = .05. Data were analyzed from October to November 2021.

## Results

**Figure 1.**
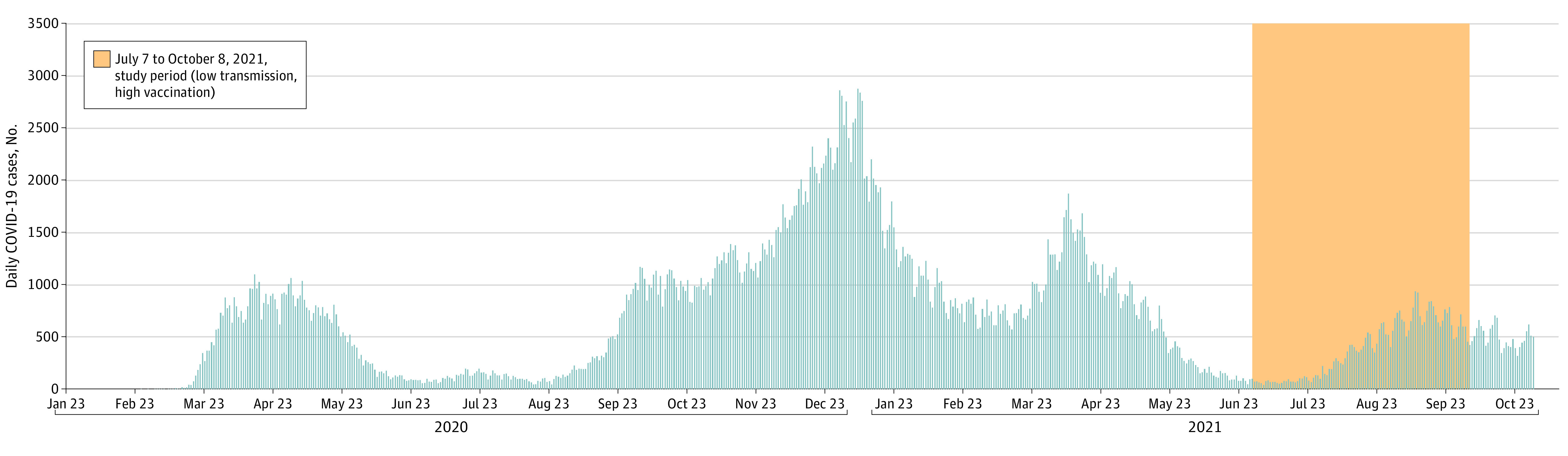
Summary Epidemiologic Context of Study Recruitment Details on business and participant recruitment are presented in Table 1.

From July 7, to October 8, 2021, 236 outbreak businesses were identified (Table 1; Figure 1); 168 businesses (71.2%) were contacted and 13 businesses (5.5%) participated. The most frequently cited reason for declining participation was a disruption to work shifts. Overall, 1892 tests were performed among 647 participants, with a mean (SD) of 2.9 (1.26) visits per participant, of which 278 participants (median [IQR] age, 43 [31-55] years; 156 [56.1%] men) had at least 1 self-testing visit, including 115 participants with 198 visits with the modified quick reference guide) (Table 2; eTable in the Supplement). RADT result was positive in 3 of 1892 tests (0.2%), of which 1 result was confirmed positive and 2 were found to be negative by PCR testing.

**Table 1.** Business and Participant Recruitment

Business or participant	No.
Businesses with outbreaks identified by Public Health	236
Businesses with outbreaks contacted by study team	168
Businesses with outbreaks visited by study team	13
Participants tested	647
Participants with ≥1 dose of any COVID-19 vaccine at time of testing, No. (%)	565 (89.1)^a^
Participants who underwent ≥1 self-test	278
Participant self-testing visits	451
Participant testing visits	
Total	1892
With positive rapid test results, No (%)	3/1892 (0.16)
Positive rapid test results confirmed by PCR, No. (%)	1/1892 (0.05)^b^

Abbreviation: PCR, polymerase chain reaction.

^a^ Among 634 of 647 participants who answered the question about vaccination status.

^b^ All positive test results underwent confirmatory PCR testing on the same day according to the study protocol.

**Table 2.** Self-reported Demographic Characteristics Among Participants With at Least 1 Self-testing Visit

Characteristic	Participants, No. (%) (N = 278)
Age	
Median (IQR), y	43 (31-55)
Not reported	14 (5.0)
Gender	
Female	109 (39.2)
Male	156 (56.1)
Do not wish to disclose	1 (0.3)
Not reported	12 (4.3)
Language preference	
English	38 (13.7)
French	227 (81.6)
Not reported	13 (4.7)
Business type	
Manufacturing	43 (15.5)
Office	196 (70.5)
Retail	27 (9.7)
Not reported	12 (4.3)
Race and ethnicity	
Asian	10 (3.6)
Black	12 (4.3)
Hispanic	14 (5.0)
Indigenous	0
Mixed	5 (1.8)
White	202 (72.7)
Other^a^	22 (7.9)
Not reported	13 (4.7)
Health conditions and habits^b^	
Chronic respiratory condition	17 (6.1)
Current cigarette smoking	32 (11.5)
Past cigarette smoking	41 (14.7)
Diabetes	12 (4.3)
Heart disease	6 (2.1)
Hypertension	17 (6.1)
None of the above	145 (52.1)
Other	16 (5.7)
Symptoms reported on the day of recruitment^c^	
Aches and pains	7 (2.5)
Chest pain or pressure	2 (0.7)
Diarrhea	2 (0.7)
Difficulty breathing or shortness of breath	4 (1.4)
Dry cough	3 (1.1)
Fatigue	20 (7.2)
Fever	1 (0.3)
Headache	12 (4.3)
Loss of smell or taste	1 (0.3)
Rash on skin or discoloration of fingers or toes	2 (0.7)
Sore throat	12 (4.3)
None	199 (71.6)
Other	2 (0.7)
Vaccination against COVID-19	237 (85.2)
1 Dose received	27 (9.7)
2 Doses received	210 (75.5)

^a^ Other self-reported race and ethnicity included Algerian, Arab, Bulgarian, Canadian, Chilean, Guatemalan, Haitian, Italian, Mediterranean, Moroccan, Peruvian, and Portuguese.

^b^ Participants could self-report 1 or more health condition. Other conditions included cystic fibrosis, Crohn disease, hyperthyroidism, irritable bowel syndrome, and allergies.

^c^ Participants could self-report 1 or more symptoms. Other symptoms included runny nose and wet cough.

**Figure 2.**
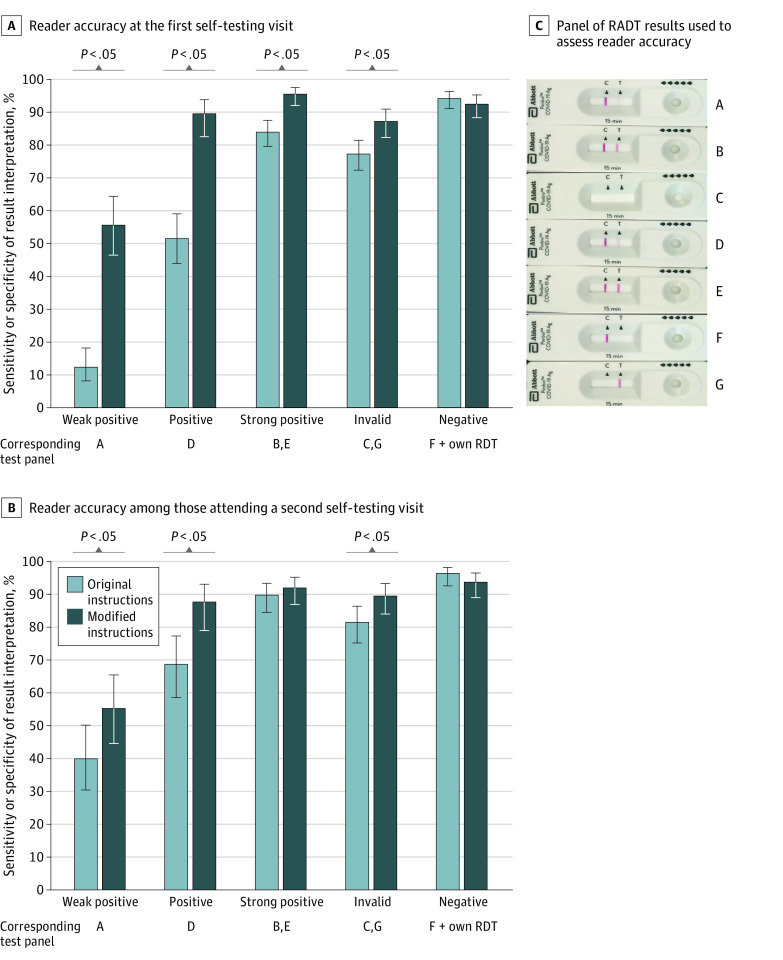
Reader Accuracy for Each Type of Rapid Antigen Detection Test (RADT) Result Interpretation, According to Instructions Provided and Reader Experience A, Reader accuracy among those with a first self-testing visit (278 participants, of whom 163 used the original version of the instructions provided by the manufacturer and 115 used the modified version designed by the investigators to address the most frequently observed errors). B, reader accuracy among those attending a second self-testing visit (173 participants, of whom 90 used the original instructions and 83 used the modified instructions). C, Panel of RDT results used to assess reader accuracy. Error bars indicate the 95% CIs for the sensitivity or specificity of the result interpretation estimated according to a binomial distribution using the Wilson Score method. Sensitivity refers to the accurate RDT result interpretations of positive and invalid results, while specificity refers to the accurate RDT result interpretation of negative results.

At the first self-test visit, significantly better accuracy in interpreting proficiency panel RADT results was observed among participants using the modified quick reference guide compared with those using the manufacturer’s instructions for results that were weak positive (64 of 115 participants [55.6%] vs 20 of 163 participants [12.3%]; difference, 43.3 [95% CI, 33.0-53.8] percentage points), positive (103 of 115 participants [89.6%] vs 84 of 163 participants [51.5%]; difference, 38.1 [95% CI, 28.5-47.5] percentage points), strong positive (219 of 229 participants [95.6%] vs 274 of 326 participants [84.0%]; difference, 11.6 [95% CI, 6.8-16.3] percentage points), and invalid (200 of 229 participants [87.3%] vs 252 of 326 participants [77.3%]; difference, 10.0 [95% CI, 3.8-16.3] percentage points). Use of the modified guide, compared with using the manufacturer’s instructions, was associated with statistically significant improvements on self-test visit 2 for results that were weak positive (46 of 83 participants [55.4%] vs 36 of 90 participants [40.0%]; difference, 15.4 [95% CI, 0.7-30.1] percentage points), positive (73 of 83 participants [87.9%] vs 62 of 90 participants [68.9%]; difference, 19.0 [95% CI, 7.2-30.9] percentage points), and invalid (149 of 166 participants [89.7%] vs 147 of 180 participants [81.7%]; difference, 8.0 [95% CI, 0.8-15.4] percentage points) (Figure 2).

**Figure 3.**
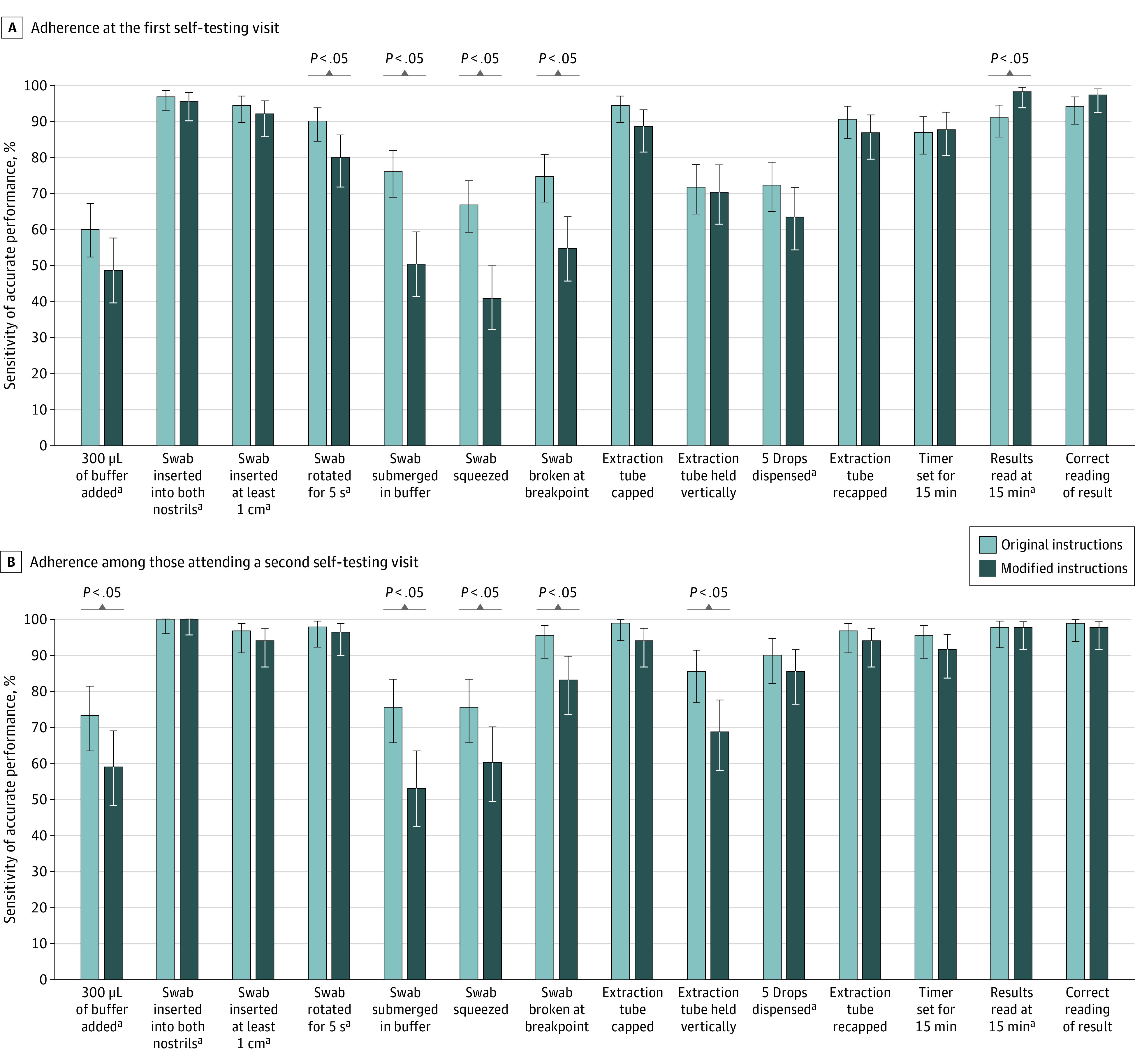
Adherence to Procedural Testing Steps, According to Instructions Provided and Reader Experience A, Adherence to procedural steps among 278 participants at the first self-testing visit. B, Adherence to procedural steps among 173 participants attending a second self-testing visit. Error bars indicate 95% CI for the proportion of participants who performed each step correctly, estimated according to a binomial distribution using the Wilson Score method. ^a^Step deemed to be most important for a valid result according to literature or expert opinion.

In multivariable models accounting for outbreak business and participant age and gender, the modified quick reference guide was associated with higher accuracy in result interpretation for self-test visit 1 (adjusted odds ratio, 13.7 [95% CI, 4.9-37.8]) but not for self-test visit 2 (adjusted odds ratio, 0.9 [95% CI, 0.1-5.5]). For procedural steps identified as critical for the validity of test results, adherence did not differ meaningfully according to instructions provided or reader experience (Figure 3).

## Discussion

In this prospective cross-sectional study of self-performed SARS-CoV-2 RADT in an intended-use setting, the accuracy of signal interpretation was proportional to test-band intensity and was extremely low for tests with weak bands. An evidence-based modified quick reference guide improved signal interpretation by untrained workers to a level of accuracy similar to that of professional technologists for other RADTs^12,13^ but was not associated with improving the proper execution of procedural steps. Repeated self-testing was associated with improved signal interpretation, although the difference was less apparent with modified instructions, as initial accuracy was already high. These results complement recent data on how consumers act on the results of at-home COVID-19 self-test kits.^14^

Self-testing using RADT has been studied for other infections. Among users attending a pretravel clinic, only 70.6% of all malaria RADT strips were correctly interpreted by untrained users.^15^ In several studies of malaria RADT, weak positive test bands consistently have correlated with lower sensitivity.^12,13,15^ Our study agrees with these findings for SARS-CoV-2 RADT, and provides evidence for a simple and effective potential corrective intervention.

It is notable that during the study period, Health Canada had not yet authorized self-performance of RADT by nonprofessionals. Because of this, the instructions for use of the RADT used in this test and all other RADT sold in Canada during this period stated for professional use only. These instructions may differ from those for the same products sold in other countries. Also, while the RADT used in this study is not available in the United States, several comparable products are sold on the US market.

Study strengths include the recruitment of participants from diverse racial and ethnic, socioeconomic, and educational backgrounds. The modified quick reference guide was informed by the most frequent or most critical errors observed and validated with different participants. Further, we assessed the independent outcomes associated with modified instructions and repeated self-testing.

### Limitations

This study has some limitations. One limitation was the small proportion of eligible businesses that entered the study. This proportion depended on several factors other than the agreement of the businesses to participate, such as our team’s capacity (ie, our four 2-person mobile teams could not always visit all the businesses that agreed to participate) and the decision not to attend businesses that agreed to participate but where the last recorded COVID-19 case was more than 14 days earlier than the day our teams could visit. Additionally, a single RADT was assessed in this study, with uncertain generalizability to the full diversity of available RADTs.

## Conclusions

This cross-sectional study found that longitudinal mass SARS-CoV-2 RADT testing could be accurately self-performed and provides evidence for a potential intervention to optimize performance. Self-administered RADTs are inexpensive and can be decentralized and implemented at scale for serial mass testing of asymptomatic persons to break chains of transmission and reduce SARS-CoV-2 incidence.^16,17^ This use case may become more pertinent with the emergence of new SARS-CoV-2 variants of concern.

## Notes

Supplement. **eFigure 1.** Serial Dilutions of the Positive Control Reagent and Corresponding Color Scale **eFigure 2.** Manufacturer’s Quick Reference Guide **eFigure 3.** Modified Intervention Instructions **eTable.** Instruction Type Used at Self-testing Visits 1 and 2
