# Supplementary material for: Adequacy of Serial Self-performed SARS-CoV-2 Rapid Antigen Detection Testing for Longitudinal Mass Screening in the Workplace
Source: JAMA Netw Open. 2022 May 6;5(5):e2210559. doi: 10.1001/jamanetworkopen.2022.10559 (PMC9077488; doi:10.1001/jamanetworkopen.2022.10559)
Supplement: Supplement. — eFigure 1. Serial Dilutions of the Positive Control Reagent and Corresponding Color Scale eFigure 2. Manufacturer’s Quick Reference Guide eFigure 3. Modified Intervention Instructions eTable. Instruction Type Used at Self-testing Visits 1 and 2 [file jamanetwopen-e2210559-s001.pdf]

Supplemental Online Content

Papenburg J, Campbell JR, Caya C, et al. Adequacy of serial self-performed SARS-CoV-2 rapid antigen detection testing for longitudinal mass screening in the workplace. *JAMA Netw Open*. 2022;5(5):e2210559. doi:10.1001/jamanetworkopen.2022.10559

eFigure 1. Serial Dilutions of the Positive Control Reagent and Corresponding Color Scale
eFigure 2. Manufacturer's Quick Reference Guide
eFigure 3. Modified Intervention Instructions
eTable. Instruction Type Used at Self-testing Visits 1 and 2

This supplemental material has been provided by the authors to give readers additional information about their work.

© 2022 Papenburg J et al. *JAMA Network Open.*

eFigure 1. Serial Dilutions of the Positive Control Reagent and Corresponding Color Scale

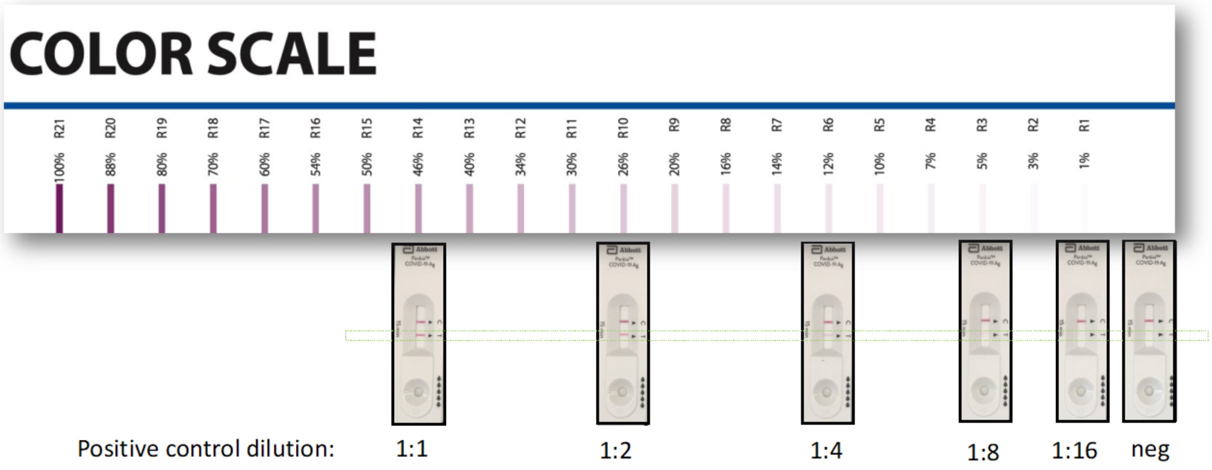

Alignment of the positive tests used to generate the evaluation panel in the present study, against a standard colour scale. The rapid diagnostic test tested span a broad range of possible signal intensities of the test line. The test line is shown in the dashed rectangle.

© 2022 Papenburg J et al. *JAMA Network Open.*

eFigure 2. Manufacturer's Quick Reference Guide

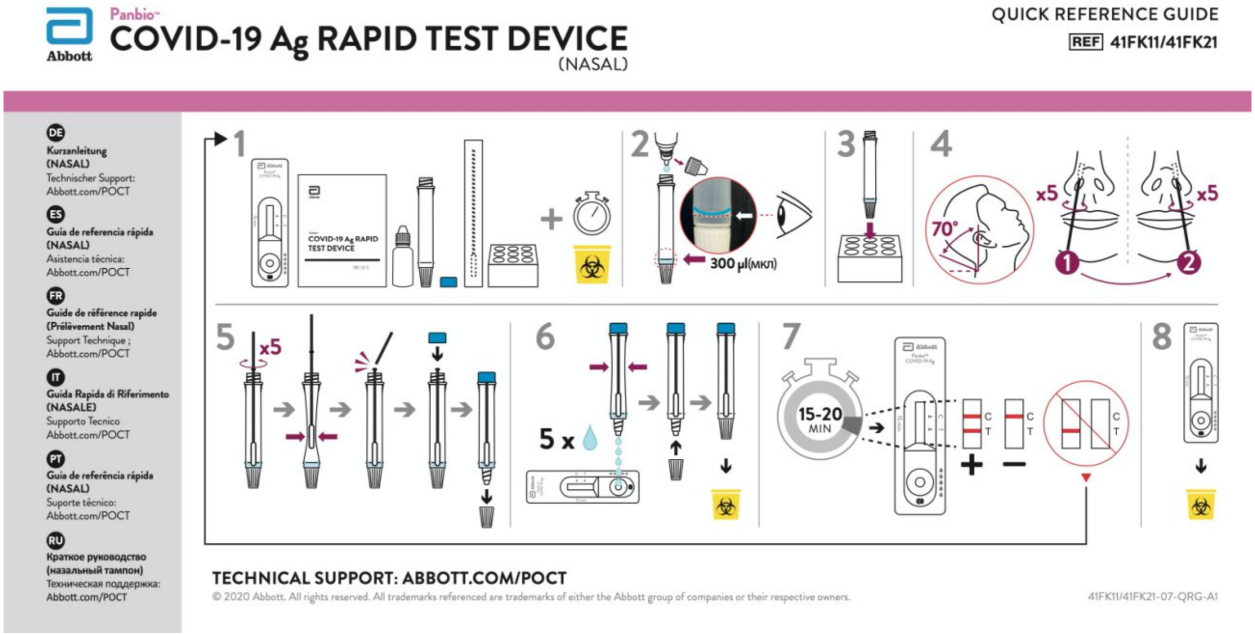

eFigure 3. Modified Intervention Instructions

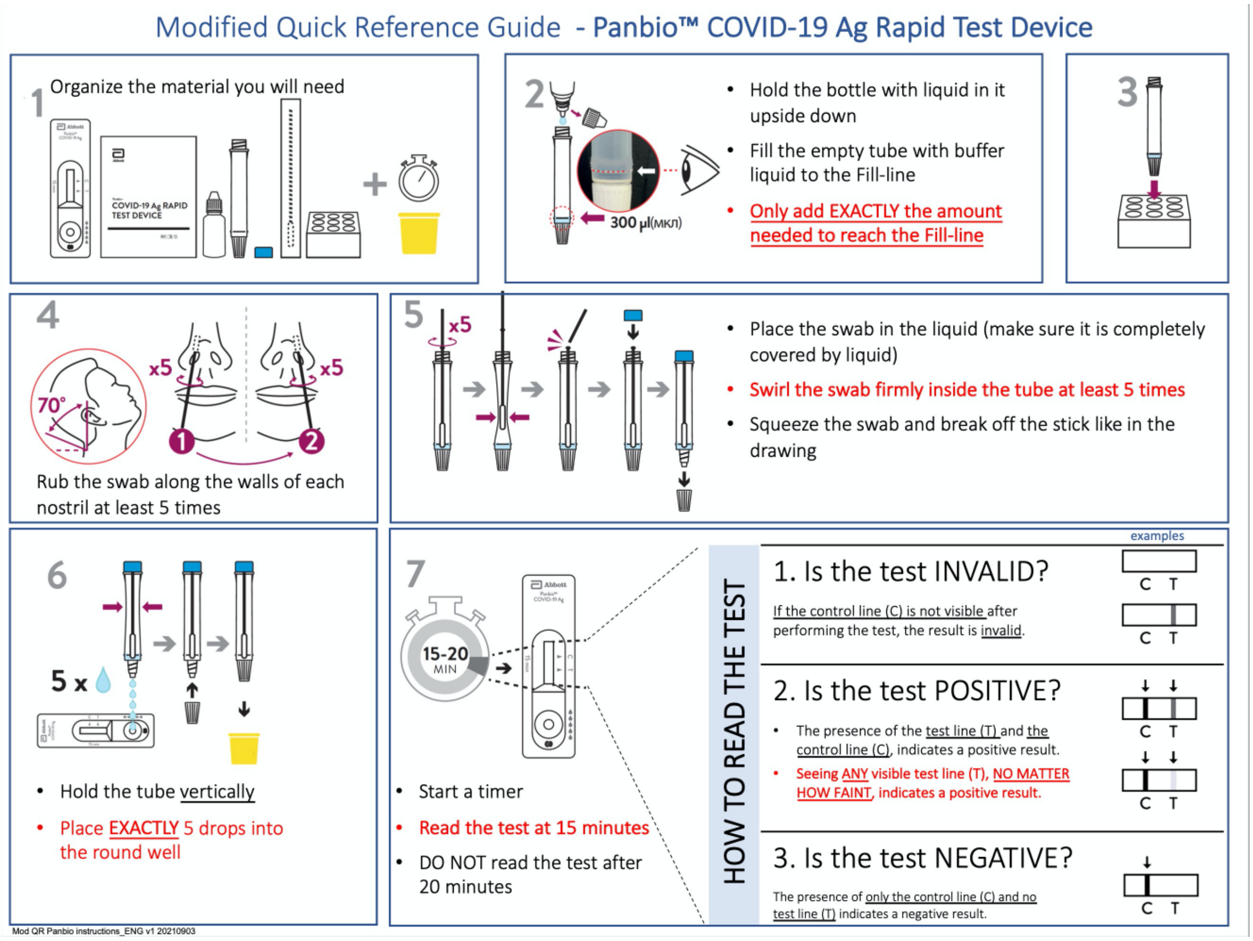

eTable. Instruction Type Used at Self-testing Visits 1 and 2

	**Self-testing visit 1 n=278**	**Self-testing visit 2 n=173**	**Total n=451**
Manufacturer's instructions used	163	90	253
Modified Quick Reference Guide used	115	83	198

The modified quick reference guide was implemented as of Sept 13, 2021.

© 2022 Papenburg J et al. *JAMA Network Open.*
